# Accessory first lumbrical muscle within the carpal tunnel: a case report

**DOI:** 10.1080/23320885.2024.2351130

**Published:** 2024-05-13

**Authors:** Yong-Seok Nam, Dong Yun Lee, Jung Soo Yoon, SooA Lim, SuRak Eo

**Affiliations:** a Department of Anatomy, Department of Anatomy, College of Medicine, Kyungpook National University, DaeGu, South Korea; b Department of Plastic and Reconstructive Surgery, DongGuk University Medical Center, Goyang, South Korea

**Keywords:** Wrist, anomalous muscle belly, lumbrical muscle, carpal tunnel syndrome, carpal tunnel release, nerve compression

## Abstract

Carpal tunnel syndrome is the most common entrapment neuropathy in the upper extremity. Palmaris longus, flexor digitorum superficialis, and lumbricals have infrequently been reported as causes of nerve compression. During routine Korean cadaver dissection, we incidentally identified an anatomic variant of first lumbrical muscle within the carpal tunnel in both wrists. The aberrant musculature originated from the radial side of the second FDS muscle at distal forearm level, running separately across the wrist beneath the flexor retinaculum. The dissected anomalous muscle was identified as an additional muscle belly of the first lumbrical muscle. Compression of the median nerve at the wrist might rarely be caused by the presence of such a tendon or muscle anomaly found in this study. Surgeons should be aware of possible anatomic variations in the carpal tunnel, and be prepared to modify their surgical plan accordingly.

## Introduction

Carpal tunnel syndrome (CTS) is rarely related to abnormal musculature within the carpal tunnel, which might increase the volume of contents within the tunnel. Since the first case of aberrant flexor digitorum superficialis (FDS) muscle of the index finger during a routine anatomical dissection reported by Mainland [1], many aberrant muscular structures in association with CTS have been described [2–6]. They often present as elongated, hypertrophic muscle bellies within the carpal tunnel.

Keese et al. [5] have classified CTS caused by aberrant muscles into two categories: 1) intratunnel intrusion of muscle belly, such as FDS [2–4], flexor digitorum profundus (FDP) [3], lumbrical muscles [6], flexor carpi radialis brevis [7], and 2) extratunnel thenar-hypothenar muscle bundles overlaying the transverse carpal ligament, such as accessory palmaris longus [8], palmaris profundus [9], abductor digiti minimi [10], and extra anomalous muscles. Anomalous muscles within the carpal tunnel act like space-occupying lesions, causing diminution of tunnel volume, which may progress to entrapment of the median nerve. Hypertrophies of those muscles that cause CTS in manual laborers and climbers have also been reported [8]. However, the frequency of nerve compressions in those cases with anatomical anomalies is still unknown.

During the cadaveric dissection, we came across an accessory first lumbrical muscle within the carpal tunnel and describe it with literature review.

## Materials and methods

**Figure 1.**
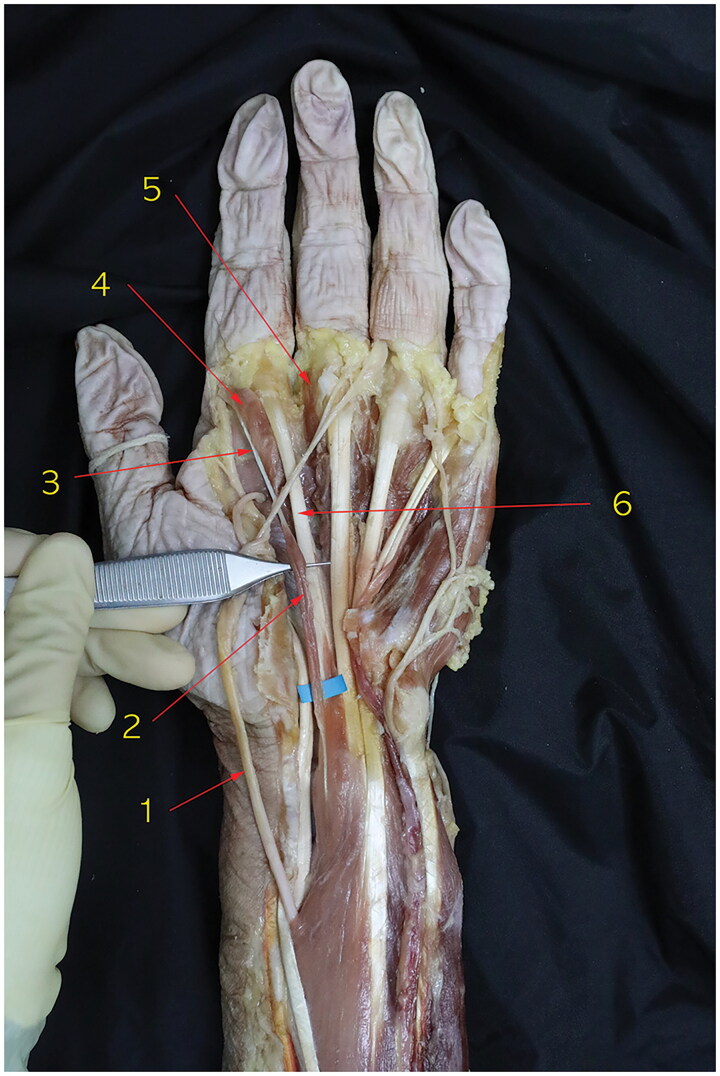
Dissection of the left wrist of an 85-year-old Korean female cadaver in the volar aspect. The anatomical structure of carpal tunnel was identified. We encountered an accessory first lumbrical muscle to the index finger within carpal tunnel (blue background). 1. Median nerve, 2. Accessory first lumbrical muscle, 3. Tendinous portion of the accessory first lumbrical muscle, 4. first lumbrical muscle, 5. Second lumbrical muscle, 6. Tendinous portion of flexor digitorum superficialis to the index finger.

**Figure 2.**
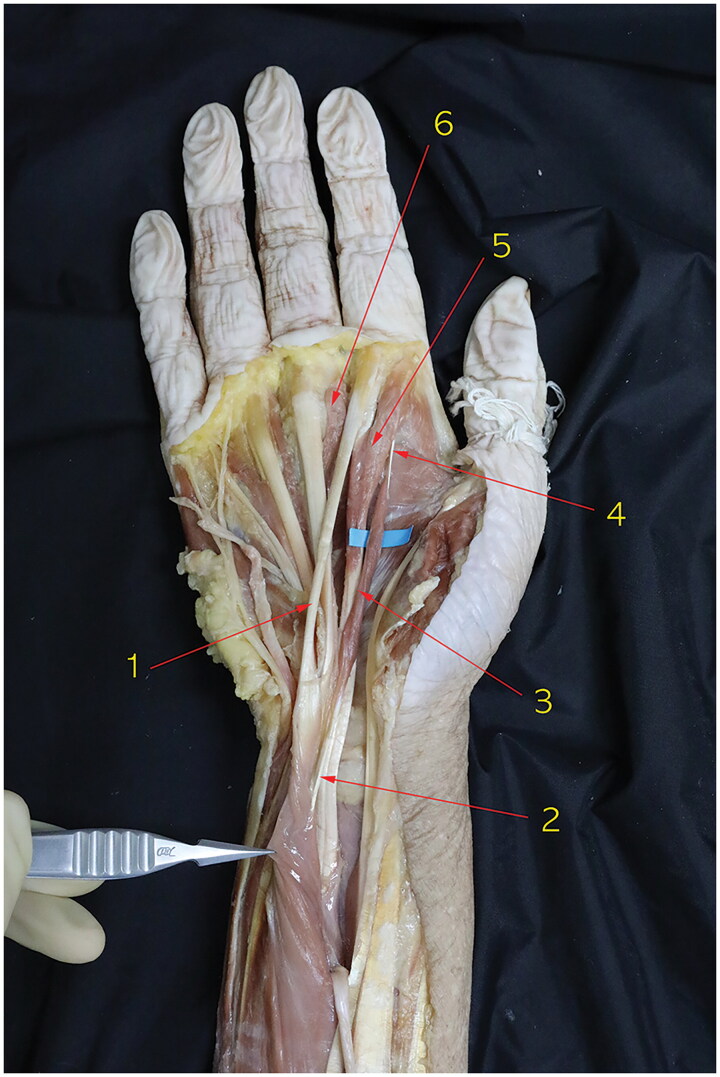
Dissection of the right wrist in the same cadaver with Figure 1. 1. Tendinous portion of flexor digitorum superficialis to the index finger, 2. Origin of the accessory first lumbrical muscle in a tendinous form, 3. Accessory first lumbrical muscle bundle, 4. Tendinous portion of the accessory first lumbrical muscle, 5. first lumbrical muscle, 6. Second lumbrical muscle.

This study was approved by the Institutional Review Board (IRB) of authors’ Clinical Research Coordinating Center (IRB number MC21EIDE0105). As a routine anatomical dissection of the cadaver of an 85-year-old female, we treated the cadaver with formalin containing 37% by weight of formaldehyde gas in water for embalming and tissue fixation. Both wrists were dissected through a volar aspect, followed extensively by forearms and hands. The anatomical structure of the carpal tunnel was identified. We noted an anomalous muscle running within the carpal tunnel in both wrists (Figures 1 and 2). There was no palpable or protruding mass before the dissection of both wrists.

## Results

**Figure 3.**
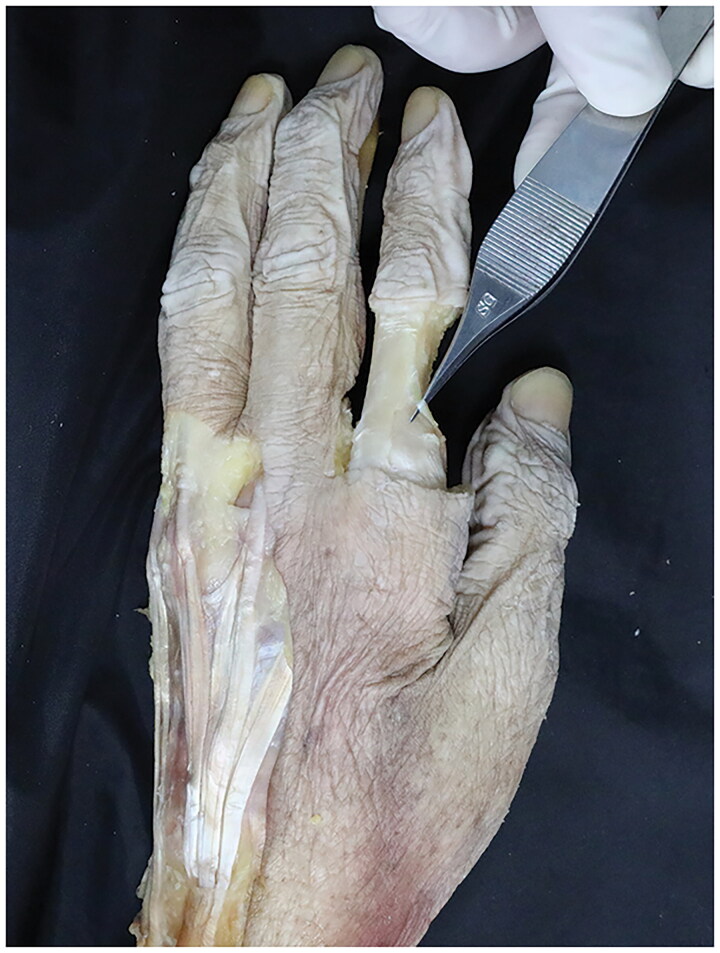
This reveals the insertion of the accessory first lumbrical muscle of the left index finger.

**Figure 4.**
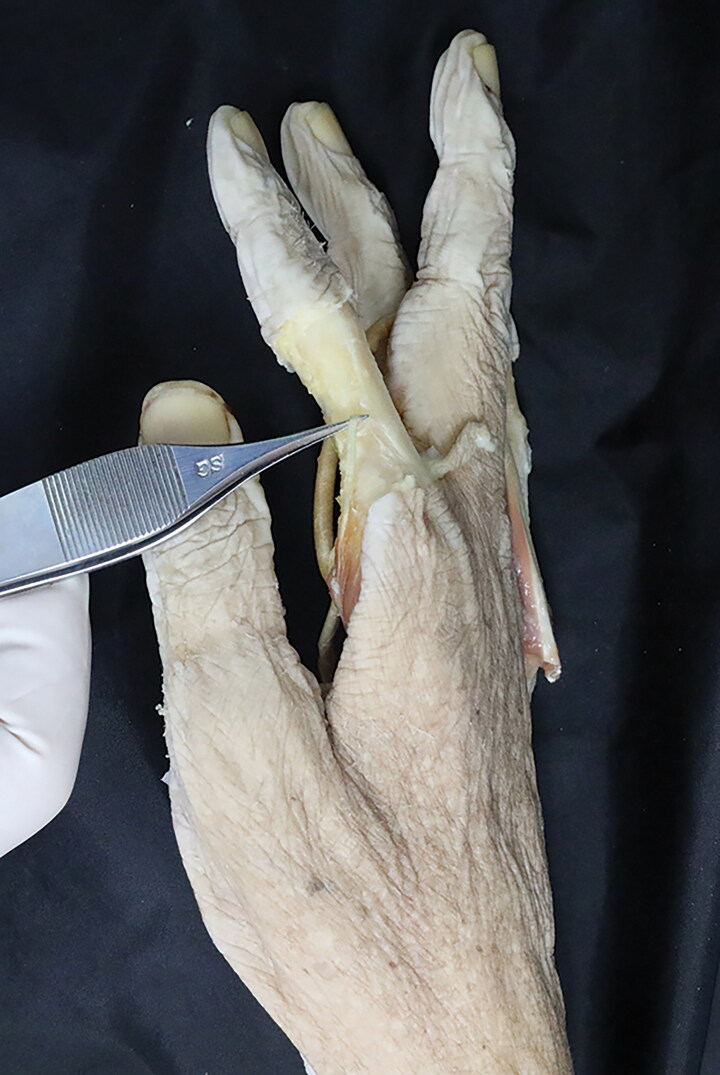
This reveals the insertion of the accessory first lumbrical muscle of the right index finger.

Within the narrowed carpal tunnel, the authors identified aberrant muscle belly along the FDS tendon to the index finger, passing radially to the median nerve. With more extended dissection, this aberrant musculature originated from the radial side of the second FDS muscle at distal forearm level, running separately from other tendons across the wrist beneath the flexor retinaculum. It changed into tendon at the palmar arch level, and continued its passage into the radial aspect of the second finger at the proximal phalanx level (Figures 3 and 4), attaching to the extensor hood mechanism of the index finger. The dissected anomalous muscle was identified as an additional extra muscle belly of lumbrical muscle into the index finger. Following elevation of the abnormal lumbrical muscle belly, the median nerve was explored just below the muscle, and released from surrounding tissues. No macroscopic evidence of compression was found in the median nerve.

## Discussion

During an open CTR procedure, hand surgeons infrequently come across aberrant small muscle bundles around the transverse carpal ligament other than thenar/hypothenar muscles, through a minimal skin incision approach.

Until now, there have been many reports of CTS associated with aberrant musculatures around the carpal tunnel [2–6]. Among them, the palmaris longus (PL) is the most variable and involved muscle in CTS [5], followed by anomalous lumbrical muscles [6], aberrant FDS [2–4], and abnormal FDP [3]. Considering that elevation of absolute hydrostatic pressures greater than 30 mmHg can induce nerve injury, the median nerve at the wrist can be influenced by muscle contraction/relaxation within the carpal tunnel and wrist posture [8].

PL loading, when loaded beyond 20 degrees of wrist extension, can increase carpal tunnel hydrostatic pressure greater than any tendon passing through the carpal tunnel [5]. Many authors have reported that it is more prevalent in a population of patients undergoing carpal tunnel release (CTR), with PL tendon being a strong independent risk factor for CTS [5,8]. In addition, its anatomical variations, such as agenesis, bifid, hypertrophic, reverse, and abnormal locations, have been reported [7–10].

Butler et al. [6] have reported the first case of an aberrant index finger lumbrical causing CTS. Abnormal flexor and lumbrical muscles bellies intruding through the carpal tunnel are the most frequent anomalies that produce a piston-like dynamic compression of the median nerve [6].

The first case of an anomalous muscle belly arising from an index finger FDS tendon was described by Smith during his procedure of CTR [2], in which he performed only freeing the muscle from the median nerve without excision. However, many surgeons currently seem to prefer excision of the encountered aberrant muscle during CTR as a viable treatment of CTS. In addition, bilateral FDS [4], FDS brevis of the little finger [5], and multiple co-existing aberrant muscle bellies within the carpal tunnel, have been also reported [6]. However, Saied et al. have found that severity or pain distribution of CTS is not related to the presence or absence of PL and FDS tendons [5], in sharp contrast to previous reports.

Rarely, the tendon of the palmaris profundus arising from the fibrous tissue of the proximal portion of the forearm can run deep to the flexor retinaculum, insert into the palmar aponeurosis, and cause CTS [9]. In addition, the flexor carpi radialis brevis muscle, which crosses the wrist and goes through the flexor carpi radialis fibro-osseous tunnel, might act as a space-occupying lesion within the carpal tunnel [7].

The unexpected appearance of the lumbrical muscle belly during cadaver dissection in our case demonstrated a highly variable anatomy of the carpal tunnel. Generally, the lumbrical muscles originate from the flexor digitorum profundus (FDP) muscles rather than the FDS muscles which our presented case revealed. This might cause some confusion in the nomenclature of this aberrant muscle. Authors designate this muscle as an accessory first lumbrical muscle. Based on our experience and the literature, we conclude that this uncommon anatomical variant should be included as a possible cause for median nerve compression within the carpal tunnel. Thus, surgeons should be aware of possible anatomic variations in the carpal tunnel, and be prepared to modify their surgical plans accordingly.
